# Paired fruit flies synchronize behavior: Uncovering social interactions in *Drosophila melanogaster*

**DOI:** 10.1371/journal.pcbi.1008230

**Published:** 2020-10-06

**Authors:** Ugne Klibaite, Joshua W. Shaevitz

**Affiliations:** 1 Princeton Neuroscience Institute, Princeton University, Princeton, New Jersey, USA; 2 Department of Physics, Princeton University, Princeton, New Jersey, USA; Santa Fe Institute, UNITED STATES

## Abstract

Social behaviors are ubiquitous and crucial to an animal’s survival and success. The behaviors an animal performs in a social setting are affected by internal factors, inputs from the environment, and interactions with others. To quantify social behaviors, we need to measure both the stochastic nature of the behavior of isolated individuals and how this behavioral repertoire changes as a function of the environment and interactions between individuals. We probed the behavior of male and female fruit flies in a circular arena as individuals and within all possible pairings. By combining measurements of the animals’ position in the arena with an unsupervised analysis of their behaviors, we define the effects of position in the environment and the presence of a partner on locomotion, grooming, singing, and other behaviors that make up an animal’s repertoire. We find that geometric context tunes behavioral preference, pairs of animals synchronize their behavioral preferences across shared trials, and paired individuals display signatures of behavioral mimicry.

## Author summary

It is often difficult to describe why an animal behaves the way it does. This is particularly true when we watch several animals at once. We can intuit from watching interactions in different species, from humans to insects, that an individual does not behave the exact same way in a social context as when completely alone. One aim of studying social behavior is to discover underlying principles that are responsible for the behaviors we observe. While we know animals communicate and engage each other, summarizing social interactions is difficult because each individual, much less each each grouping of animals, is different. Here we measure the interactions of paired fruit flies in a systematic way, and capture how different social pairings affect the behavior of the animals. We find males and females pay attention to their environments and what their partners do at different distances, and paired individuals tend to synchronize behavior with their partners across all types of pairings.

## Introduction

Social behaviors are exhibited by a wide variety of species and include such diverse categories as courtship, aggression, dominance, collective flocking, and group decision making [1–10]. Ultimately, the actions an animal performs are influenced by a combination of genetics, environment, social pressure, and internal state [11–13]. Separating these contributions to isolate the nature of social interaction has remained a challenge. It is often difficult to define precisely what a social behavior is. Studies of social behavior range from characterizing simple metrics such as distance between individuals [14, 15] to machine learning methods aimed at identifying and scoring the occurrence of specific behaviors from human-annotated datasets [16] to studies of the emergent properties from the social interactions of large numbers of animals in a flock or school [17]. Here, we focus on the effect of one individual on another’s behavior in a paired context.

In the past, proximity data has been used to discover rules governing the short-range interactions between individuals in groups [15, 18, 19]. Other work looked unidirectionally at the effect of one individual’s locomotion on sensory stimuli from a partner [20–23]. Social behavior is bidirectional, and these previous methods are insufficient for studying the reciprocal nature of social behavior and are not easily extended to behaviors that are more complicated than locomotion. We build on this previous work by recording the position and full behavioral repertoire of pairs of fruit flies, and separately quantify the effects of environment and social interactions on an individual’s behavior [24].

**Fig 1:**
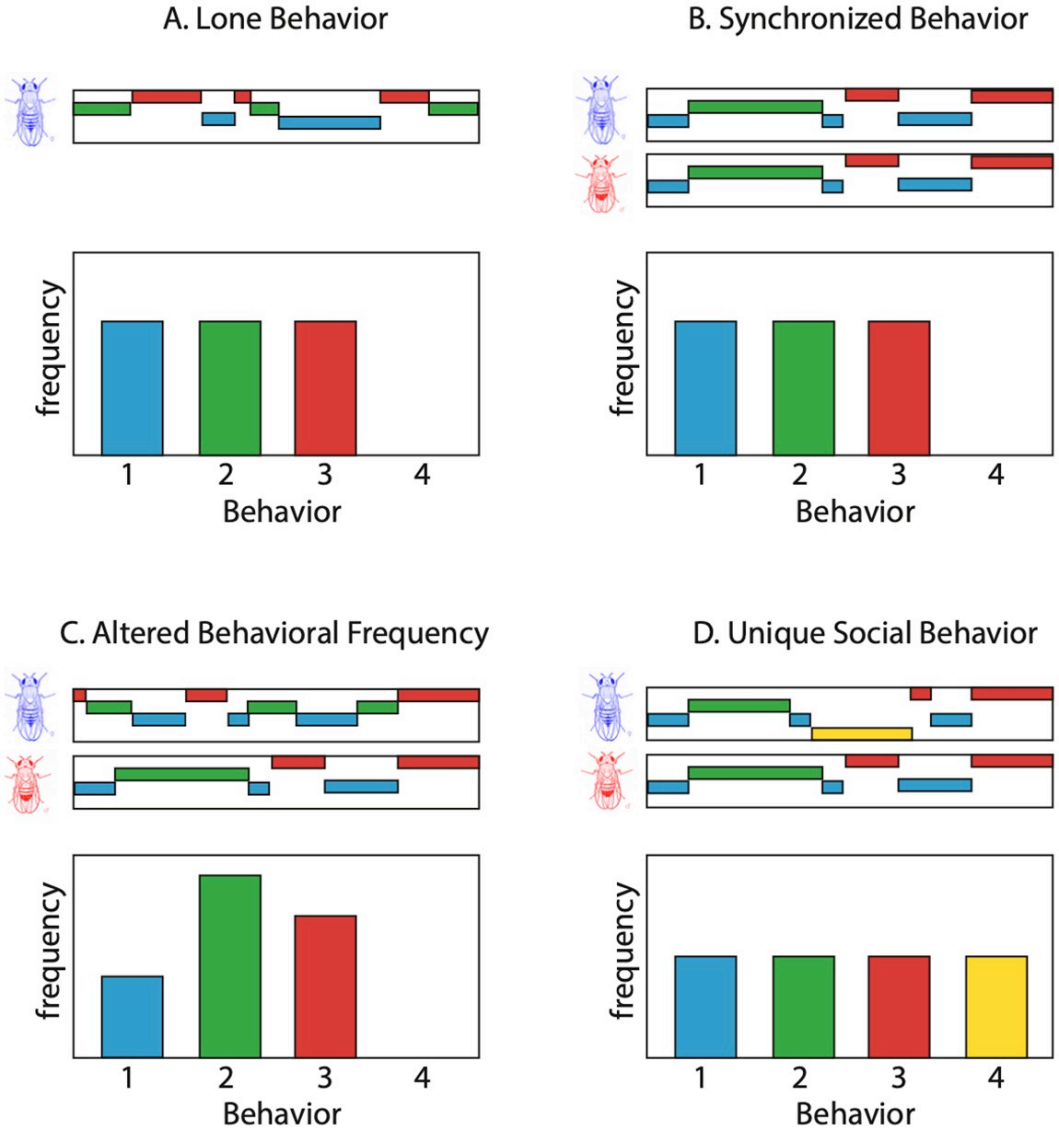
A schematic of ways in which individuals may alter behavior during social interaction. (A) A hypothetical ethogram and behavioral density for a lone fly (blue). (B) Ethograms and behavioral frequency in the presence of a social partner when the two flies synchronize their behaviors in time. (C) The presence of the second fly alters the frequency of the different behaviors without synchronization. (D) A new social behavior (#4) arises in the presence of the partner.

Social behaviors can manifest in multiple ways as illustrated in Fig 1. We represent schematically the behavior of a hypothetical fly through its behavioral time series, or ethogram, and a histogram of the relative frequencies of the occurrence of each of four behaviors. This individual (shown in blue) performs three behaviors, switching stochastically between them, but overall performs each behavior for the same total amount of time as seen in the bar plot (Fig 1A). The next three panels deal with three possible expressions of social interaction when a second fly (shown in red) is added to the arena. In one scenario, the overall time spent performing each behavior is unaltered but the two flies synchronize their behaviors in time as can be seen in the ethograms (Fig 1B). Alternatively, the presence of the red fly could cause the blue fly to alter the time spent in each behavior (Fig 1C). Finally, we consider the possibility that the presence of a partner causes the fly to perform a fourth behavior that only occurs in a social context (Fig 1D). We looked for signatures of these possibilities in our data and find evidence for each type of interaction.

The simultaneous performance of the same behavior in con-specifics (Fig 1B) has been reported across many species, and the phenomenon has been referred to as mimicry (not to be confused with Batesian mimicry, where one species copies the physical appearance of another [26]), imitation, synchrony, and contagion [25, 26]. Neural mechanisms of behavioral mimicry have been explored in humans and the discovery of mirror neurons, cells which reliably fire during the execution of motor sequences performed by others, has spurred hypotheses about their mechanisms in learning and pro-social behavior [27]. The proposed functional roles of mirror neurons in humans and certain apes include action understanding and action learning, properties which are not likely to exist in simpler organisms [28]. However, even simple behavioral mimicry can benefit a group of individuals, and provides evolutionary advantages such as enhanced avoidance of a predator and the facilitation of feeding behaviors [29–34]. Social facilitation has also been shown in fruit fly grooming, and suggests that grooming may play a part in social signalling [35]. These effects can arise from simple behavioral matching, where an animal performs a behavior already present in its repertoire in response to the action of another, and do not require complex cognitive mechanisms [36, 37]. More recent work has shown that fly dyads separated by a translucent barrier exhibit correlations in their distances to the barrier, and that freely interacting pairs of flies align several other behavioral features such as circling and wing usage [38, 39].

Here, we ask whether there are discernible behavioral effects arising from simple social pairings of fruit flies by using an unsupervised behavioral quantification paradigm. We characterize behavior directly from movie images in the same way regardless of social context, and use the resulting behavioral labels to investigate how individuals regulate behavior when placed in different pairings. Our analysis tracks the occurrence of a broad set of behaviors including grooming, locomotion, crawling, and idle behaviors. These categories are defined via an unsupervised clustering method followed by human annotation [24, 40].

Comparing the effects of same-sex and courtship pairings on behavior, we find that the behaviors performed by an individual depend on not only the social pairing but also the spatial setting an individual finds itself in. We characterize the behavioral effects induced by the location of a fly within the experimental arena and the distance of the individual to its interaction partner, and identify the presence of behavioral mimicry within interaction pairs at short time scales.

## Materials and methods

### Behavioral movie recordings

We used a custom-built rig to film the behavior of interacting fruit flies. Our rig can accommodate four experiments at once, allowing for the acquisition of two dozen half-hour movies per day. The setup is the same as described in [24], where four cameras are used at once to record the activity in four separate domes placed on the same back-light. Flies are loaded by gentle manual aspiration and movies are started within a minute after flies are introduced into the arena. Because fruit fly activity and courtship levels are known to vary over their circadian cycles and lifetimes, we aim to capture behavior from flies at similar times within these cycles and film only within the four hours after the lights come on. To keep experiments consistent, we isolate flies upon eclosion and age them four to six days before imaging.

In order to address the effects of a paired social context, and not just the courtship context, we filmed behavioral movies of male-female pairs as well as male and female same-sex pairs. Additionally, movies of isolated flies from the same population for either sex provide a control that allows us to compare spontaneous behavior to interaction. The number of movies and time of behavior recorded for each pairing is summarized in Table 1. Courtship movies are not analyzed past the point of successful copulation, and therefore have variables lengths, whereas all other movies are analyzed over the entire length of recording.

**Table 1:** Number of movies recorded, total recording time, and number of frames analyzed are listed for each behavioral context. All analyses were performed on all data described here.

Behavioral Movies			
Social Context	Number of Movies	Total Time (hr)	Total Frames
Courtship (Female)	152	38.1	13,710,669
Courtship (Male)	152	38.1	13,710,669
Female-Female	81	73.1	13,155,080
Male-Male	70	63.9	11,501,343
Lone Female	61	30.5	10,966,897
Lone Male	55	27.5	9,888,414

### Behavioral analysis

We performed behavioral analysis for all recorded movies using the pipeline introduced in [40]. Table 2 lists the parameters used for image PCA, wavelet decompositions, tSNE embedding, and clustering. The original embedding was performed using a sampling of points from all movies in order to facilitate comparisons between the various social contexts. As the total data set is too large to store the relevant distance matrix in memory (about 60 million data points), we instead train the tSNE algorithm on a smaller training data set of *N*_train_ = 38,500 points. To generate an informative training set, we first perform a tSNE embedding of regularly spaced points for each movie in the dataset and then build a training set from a diverse selection of points from each movie. This training set was used to perform a final tSNE embedding into which each movie was re-embedded as described previously [40]). We then applied a watershed transform to the resulting density histogram to delineate clusters with local density peaks (for more information refer to [40]).

**Table 2:** Parameters and values used in the behavioral analysis pipeline. For more details see the supplement of [40].

Pipeline Parameters of Interest		
Parameter	Value	Description
*N*_*θ*_	90	Number of angles used in Radon transform
*M*	50	Number of postural eigenmodes used for projections
*N*_*f*_	25	Number of frequency channels used in wavelet decomposition
*N*_train_	38,500	Training set size after subsampling
*ω*	1.5	Width of Gaussian kernel used for generating density map

Movies sampled from each category of behavior were manually inspected and found to fall into eight broad categories (Table 3). Examples from several clusters are demonstrated in Supplemental S1 and S4 Videos. S1 and S2 Videos show specific wing movements that were labeled ‘left wing song’ and ‘right wing song’ and then combined under the broad category of ‘song’ in our manual clustering step.

**Table 3:** Eight coarse behavioral clusters were used to describe 116 fine-grained behaviors obtained from the behavioral map. These broad descriptions were used to sort behaviors and label the behavioral map.

Descriptions of Behavioral Groups	
Coarse Label	Description
Song	Wing vibrations used by males during courtship, identified by extension of the wing at an approximately 90 degree angle from the body
Wing	Movements involving grooming the wing or flutters of the wing that do not constitute full extension
Locomotion	Directed movement that involves limb coordination
Crawl	Slow and uncoordinated locomotion that does not appear regular
Anterior	Rubbing of the head and front legs
Posterior	Rubbing of the hind legs
Small/Slow	Short or slow movements that occur when an individual is otherwise still, often constituting of an extension of a single limb
Idle	No discernible movement

### Calculating behavior correlations

Probability densities for each coarse behavior are found by calculating the fraction of time an individual spent in each state as determined by embedding in the behavioral map. The densities for any given individual or experimental group can be written as *P* = [*p*_*song*_ + *p*_*wing*_ + *p*_*locomotion*_…] where each entry of *P* is the fraction of time spent in the given behavior, and the sum over *P* is one. Correlations between behavioral probabilities within individuals and between paired individuals are measured using the Pearson’s linear correlation coefficient. For example, the correlation between male song behavior and female locomotion in the courtship case is calculated by finding the Pearson’s correlation coefficient of all male courtship *p*_*song*_ probabilities with their corresponding female courtship partners’ *p*_*locomotion*_ probabilities. This returns a measure of how well each pair of behaviors is correlated between social partners irrespective of timing.

### Density map similarity metrics

We quantify the similarity between experiments in different social contexts by calculating the Jenson-Shannon (JS) divergence between their behavioral probability distributions (Eq 1) [41]. Since any behavioral map (represented by a 501x501 matrix) may have very small values, a mask is first applied to each map before comparison so that only entries with value > 1*E* − 6 in the mean behavioral map are used. All infinite and NaN values are then removed before summation. Units are presented in bits.

$$\begin{array}{c} JS(P∥Q)=\frac{1}{2}D_{KL}(P∥M)+\frac{1}{2}D_{KL}(Q∥M)\;\text{where}\;M=\frac{1}{2}(P+Q) \end{array}$$ (1)

$$\begin{array}{c} D_{KL}(P∥Q)=-\sum_{i}P\left(i\right)\log\frac{Q(i)}{P(i)} \end{array}$$ (2)

### Mutual information analysis

In order to probe the temporal coupling of behaviors, we calculate the mutual information (MI) between paired behaviors for all movies in a given context and for each movie individually. We use the following equation, where *B*_1_ and *B*_2_ are the underlying distributions of behaviors for a given type of individual.

$$\begin{array}{c} MI\left(B_{1};B_{2}\right)=\sum_{b_{1}\in B_{1}}\sum_{b_{2}\in B_{2}}p\left(b_{1},b_{2}\right)\log(\frac{p(b_{1},b_{2})}{p\left(b_{1}\right)p\left(b_{2}\right)}) \end{array}$$ (3)

While this calculation provides a measure of how much information is present between two behavioral time series of interest, it is more informative to consider the pointwise entries of the MI before summation, which is calculated for each pairwise set of behaviors as $p\left(b_{1},b_{2}\right)\log(\frac{p(b_{1},b_{2})}{p\left(b_{1}\right)p\left(b_{2}\right)})$. This value reveals how much each pairwise entry contributes to the total MI, and accounts for how often a pair of behaviors co-occur compared to their expected co-occurrence assuming no social interaction. These values may be negative if behaviors occur together less than expected assuming independence.

Given the large and variable sizes of our datasets, it is difficult to interpret the absolute value of the MI. We thus produced synthetic data using an 8-state Markov Model (MM) with probability and transition matrices matching our data from trajectories through the coarse behavioral space for each interaction context. This synthetic data is the same length as the true data for each context and provides a baseline level for the MI expected from a random Markovian process that can be compared to the MI from experiments.

## Results

We recorded the behavior of *Drosophila melanogaster* across lone and paired individuals in a featureless circular arena of radius *R*_arena_ = 11 mm for up to 30 minutes (or until copulation in the case of courtship pairings). The number of experiments and total time recorded for each pairing are summarized in Table 1. Video was recorded from above on a back-lit stage, where flies were allowed to freely move and interact under a plastic dome. The resulting videos were analyzed using an unsupervised behavioral platform to assign behavioral labels [24, 40]. We also extracted the position and orientation of each individual over time from the videos.

### Position and orientation for single and paired flies

**Fig 2:**
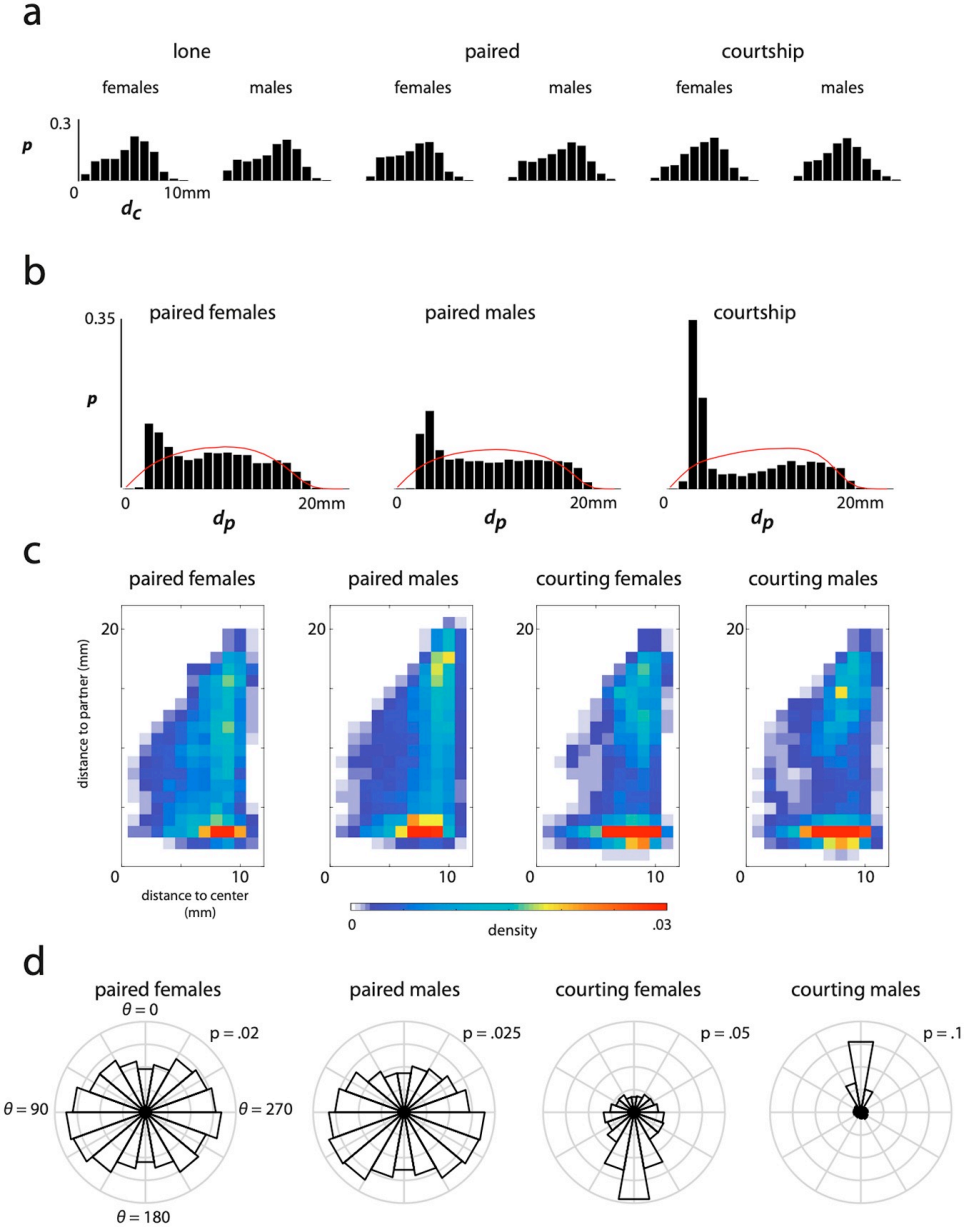
Distance and angle within the arena and to partner by behavioral context. Histograms of distance to the center of the arena, distance to partner, and heading to partner are plotted where applicable. a) The fraction of time individuals from each context reside in ten equal-area concentric circles radiating from the center of the arena outwards. b) Histogram of the distance between paired individuals for all individuals in a given context. The red line indicates the expected distribution of distances between random points within the arena. c) 2-dimensional histogram of residence time in each paired context broken down by distance from the center of the arena (x-axis) and distance to the interaction partner (y-axis). d) Radial histogram, *p*(*θ*), of the heading of an individual given a context in relation to the centroid of the arena partner where *θ* = 0 refers to heading directly toward the interaction partner. Bins each represent a 20° angular range. The value of the outer ring is labeled in each radial histogram as they are scaled differently.

The shape and size of the physical environment can affect where animals go and what they do. To start, we tracked the position and orientation of each animal in the arena across all behavioral recordings. Flies in all measured conditions showed a preference for the edge compared to the middle of the arena, which we quantified through the distance to the center, *d*_*c*_ (Fig 2A). This effect has been previously reported when flies are restricted to a circular arena, and shows the importance of arena geometry when considering spontaneous behavior [42]. We also see a drop off at very high *d*_*c*_ as the fly must push itself into the sloped region of the domed arena to occupy those locations.

For paired contexts, we calculated the distance between the individuals, *d*_*p*_ (Fig 2b). These distributions are bi-modal for all pairings with a narrow peak at short distances and a broader tail that extends to the largest separation that the animals can achieve, *d*_*p*_ = 2*R*_arena_. These features are indicative of an interaction between the animals and not consistent with a model in which the position of the animals is uncorrelated. We compared the histograms in Fig 2b to the expected distribution of distances between two points randomly chosen from the measured probability distributions of occupancy in the arena (red lines) and find that all pairings spend more time at short distances than for this null model.

We further find that individuals in courting pairs spend much more time close to each other than those in same-sex pairs. This is likely because most of the behaviors associated with courtship occur at a short distance to allow for physical, chemical, and auditory communication and attempted copulation. Interestingly, the male-male and female-female pairs also exhibit a peak at short distances, indicating that these pairings also induce short-range social interactions. To further visualize the combined effects of pairing and the environment, we plotted the two-dimensional histogram, *P*(*d*_*c*_, *d*_*p*_), for each context (Fig 2C). For same sex pairs, the short-range interactions mostly occur near the edges of the arena (high *d*_*c*_) whereas courting pairs display short-range interactions over a larger area in the arena.

We next examined the preference of flies to orient themselves relative to a partner (Fig 2D). We define the angle of heading for a paired individual as the displacement in degrees from facing toward the interaction partner as described in [24]. The courting male predictably spends most of his time facing toward the female while the female is unlikely to face toward the male during courtship as she spends most of the time moving away from the male. The same-sex pairings exhibit more uniform distributions of heading, indicating that they do not prefer to face towards or away from their interaction partner as strongly as during courtship. Interestingly, the paired males and paired females show a suppression of heading directly toward their interaction partner. This effect has been suggested previously, when it was shown that a female odor incited males to orient toward and touch other flies, but a male odor had a lesser effect [43].

### Quantification of behavior across social contexts

**Fig 3:**
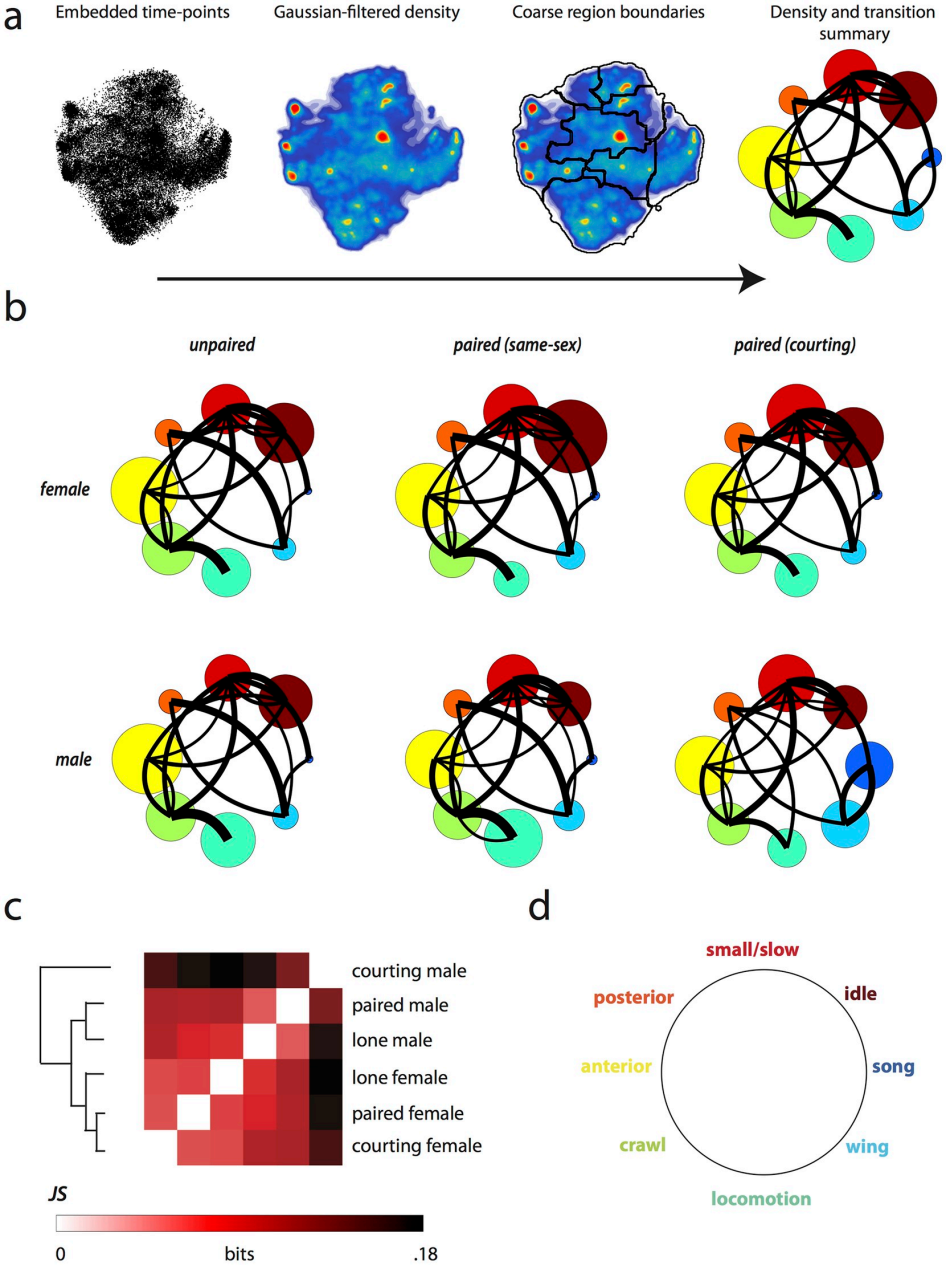
Behavioral densities across paired and lone contexts. a) Representative images from each of four steps used to visualize behavioral frequency. In sequence the images describe the progression of time-points on the two-dimensional behavioral space, the Gaussian filtered behavioral density, boundaries for coarse-grained regions based on hand-labeling, and finally the bubble plot summarizing the relative densities and transitions between coarse behavior regions. The size of each bubble represents the relative amount of time spent in that behavior and black lines show the transition probabilities between behaviors with line thicknesses proportional to the probability and right-handed curvature representing the direction of transition. b) Coarse behavioral densities for each of the six categories of experiment. c) We compare the densities across the six conditions by visualizing the JS divergence (Eq 1) across all groups, ordering the rows and columns based on similarity. d) Manually assigned labels describe the eight broad categories of behavior exhibited during the behavioral movies with color and location corresponding to the circle density plots.

We quantified the behaviors exhibited by lone and paired flies using the method described in [40] and [24]. In summary, each time point from a movie is mapped to a point in a two-dimensional representation of the postural dynamics of the body (Fig 3). The estimated two-dimensional probability density, visualized as a heat map such as in Fig 3a, describes the behavioral repertoire of a set of individuals. Clusters from the two-dimensional point cloud, found using a watershed algorithm on the estimated density, represent distinct stereotyped behaviors. A watershed transformation of this density generated from all recorded individuals produces 116 fine-grained behavioral clusters that capture all of the behaviors performed by lone and paired flies in our experimental arena. We manually assigned each of these clusters to one of 8 coarse-grained behavioral labels (song, wing, locomotion, crawl, anterior, posterior, small/slow, and idle) by viewing randomly selected movie clips that were assigned to each fine-grained cluster. Fig 3a shows the progression from points in the two-dimensional space to a density map which is then segmented based on the coarse labels. Finally, we visualize the frequency of each behavior in a bubble plot where the area of each circle corresponds to the relative amount of time a given behavior was performed. The colors and position for each label are shown in Fig 3d. The transition probabilities between pairs of behaviors are displayed using curved black lines [44]. These plots (Fig 3b) are then used to compare the relative frequency of each behavior in a given context, as well as to show the transition structure between the coarse-grained behaviors.

We find that behavior varies based on social context (lone, or paired with a same-sex partner or opposite-sex courtship partner, Fig 3b). Males perform more fast locomotion behaviors than females when isolated in the experimental chamber, while females tend to crawl and turn more often. Lone individuals of both sexes perform anterior grooming more than their paired counterparts, but paired individuals perform more posterior grooming and wing movements across all pairings. Females in a same-sex pairing are idle much more often than individuals in any other experimental group. Individuals in any of the paired contexts display an increase in small body movements (such as reaching using the legs) over isolated individuals. Locomotion decreases from the lone baseline in each paired context except for male-male pairings, where there is a sharp increase. Finally, male individuals in same-sex pairings also display a decrease in idle behaviors over their lone counterparts.

To compare behavior quantitatively, we calculated the Jensen-Shannon (JS) divergence between the behavioral probability densities for all pairs of contexts (Eq 1, Fig 3c) [41]. The JS divergence measures the similarity between two probability distributions. By performing a hierarchical clustering of all pairwise similarities, we find that the three female contexts are most similar to each other, followed by the lone and male-male paired contexts. Courting males are by far the most different, driven by courtship-specific song and wing behaviors. Interestingly, males that are lone or paired with another male are more similar behaviorally to females from all contexts than to courting males.

### The effect of arena position on behavior

**Fig 4:**
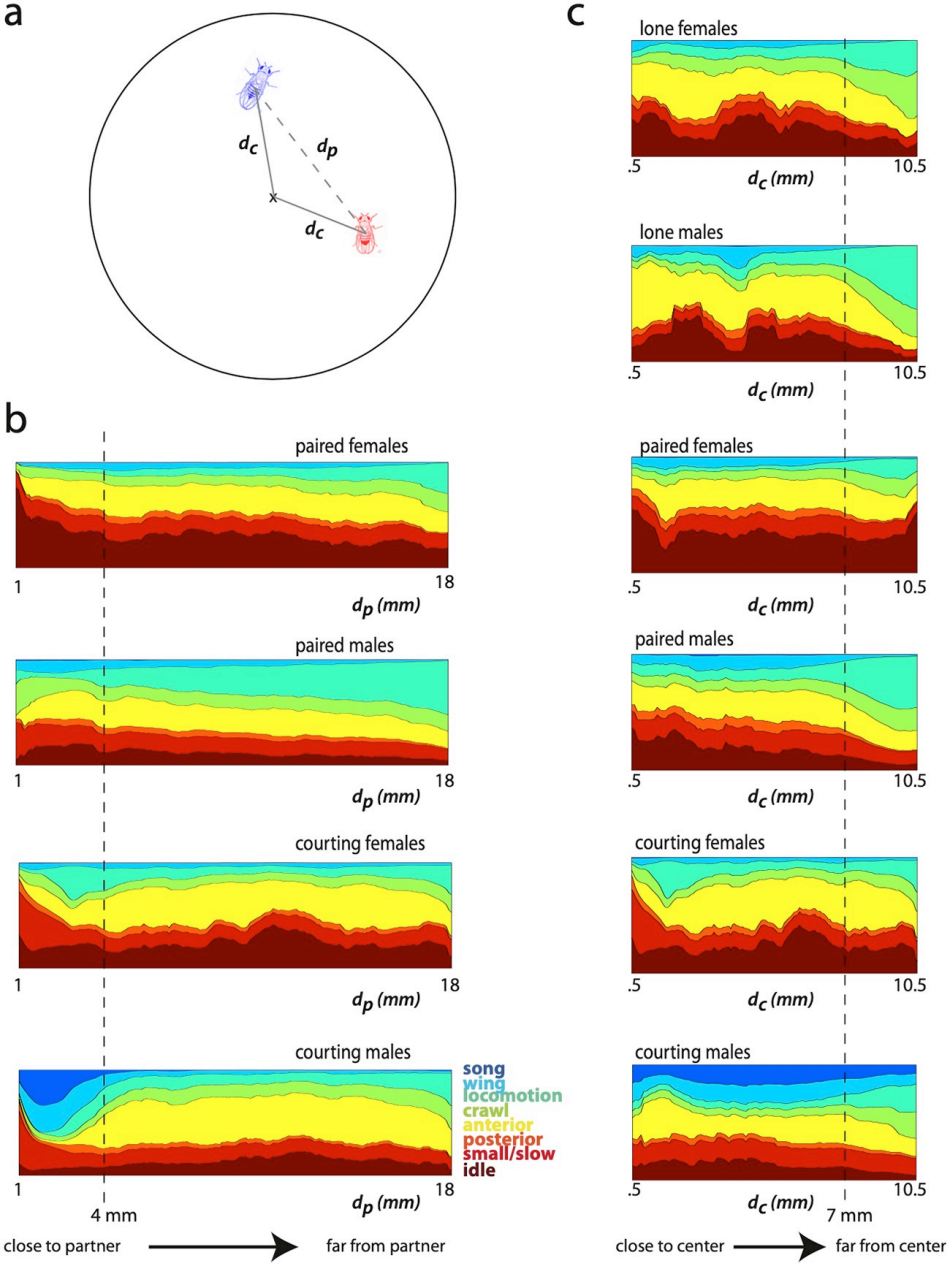
Fly behavior by radial position in the arena across context. a) Illustration of distances *d*_*p*_ and *d*_*c*_ given a pair of flies in the arena. b) Stacked behavioral density plots show the frequency of each coarse behavior given distance to the interaction partner *d*_*p*_. A sliding window of 1 mm was used to calculate density at.1 mm increments with centers ranging from.1 to 18 mm. A stacked plot is shown for each paired condition. Color corresponds to the coarse behavior labels. c) Stacked behavioral density plots show the frequency of each coarse behavior binned in 1 mm radial windows centered at the distance *d*_*c*_ from .5 to 7 mm. A stacked plot is shown for each experimental condition, where color corresponds to behavioral frequency at each radial position in the experimental arena. The dashed line indicates a radial distance of 7 mm.

We combined measurements of animal position and postural dynamics to investigate the effect of environment on behavior. We visualized the behavior of individuals from each experimental group at varying distance from the center of the arena (Fig 4c). First, we considered the behavior of lone individuals given arena position and found that active behaviors such as locomotion and crawling increase the further an individual resides from the center of the arena, as has previously been reported [42]. Males in particular spend a large portion of time circling the arena, illustrated by the sharp increase in locomotion fraction after the 7 mm mark. Anterior, posterior and wing grooming, on the other hand, are more commonly performed toward the center of the arena and suppressed at the edge when compared to locomotion and crawling.

**Fig 5:**
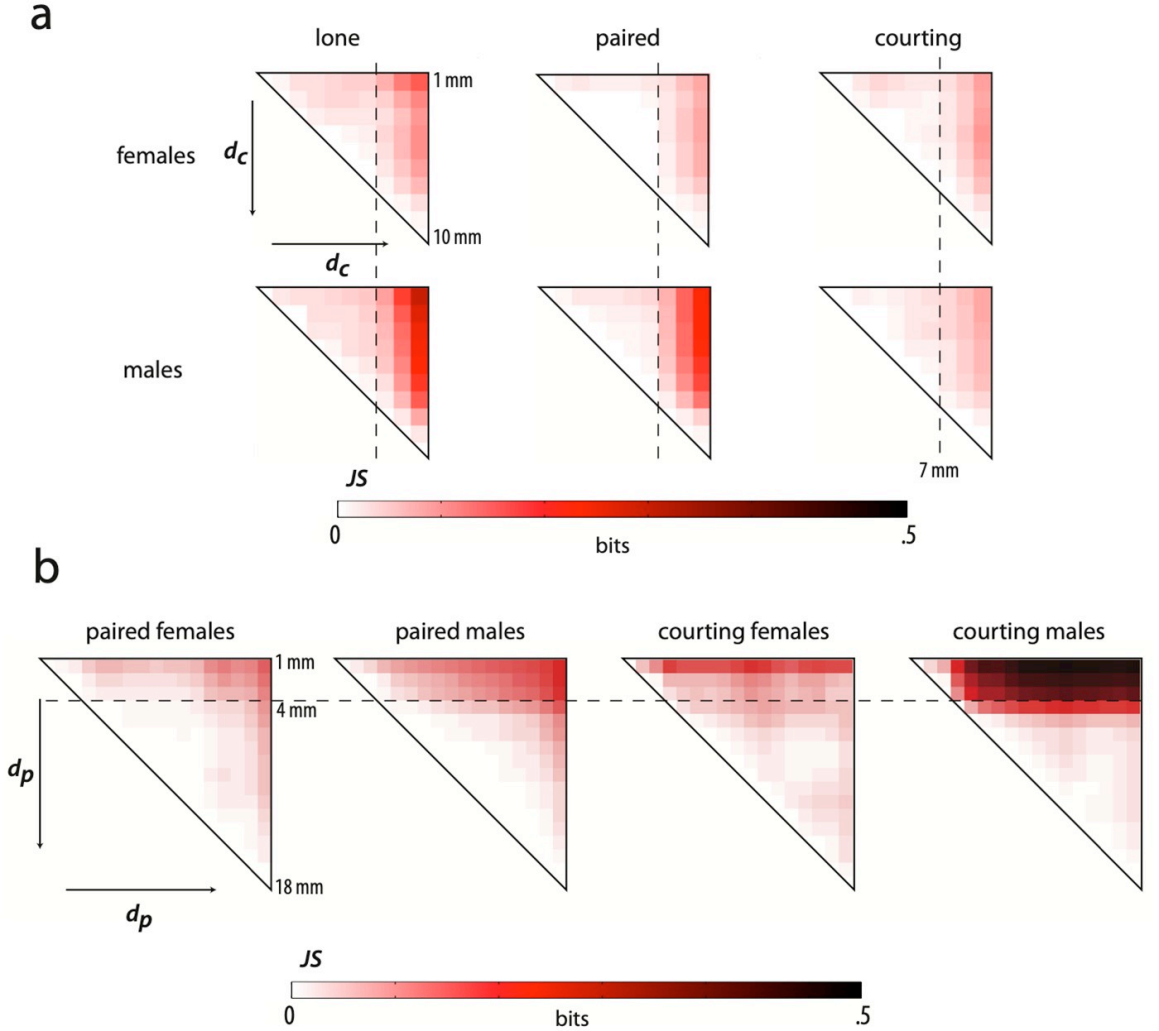
Changes in overall behavior as a function of distance to the arena center and the partner. a) Comparison of behavioral density maps generated for each context given 1 mm bins of distance to the center of the arena. A behavioral map is generated for each context given the behavior of individuals at a specific 1 mm window of distance to the center of the arena. The JS divergence is computed between each map within a context, and the upper right section of the resulting matrix is displayed. The dashed line in each matrix indicates a radial distance of 7 mm b) A comparison of density maps for overlapping 2 mm bins of distance to partner is generated in the same manner as in (a) and the JS divergence between each map in a given context is displayed. The dashed line in each matrix indicates a partner distance of 4 mm.

Paired flies show the same trend as a function of distance from the arena center, and we see enriched locomotion at the edges of the arena (Fig 4c). Behavior does not depend linearly on distance from the center of the arena. We quantified the amount of change in behavior with position by computing the JS divergence between behavioral densities at different radii (Fig 5A). For all contexts, the distribution of behaviors observed from time points where flies are within 7 mm of the center are fairly similar whereas there is a marked change in behavior at the 7 mm mark. This effect is stronger in males than females, and is not as prominent in the courtship condition for either sex, likely due to social arousal interfering with environmental cues.

### The effect of a partner on behavior

We have previously shown that distance to a courtship partner has an effect on behavioral density [24]. We generated behavioral maps for each paired context from time points spent in sliding 2 mm distance bins to the partner, and find that this is also true for same-sex pairings. We performed the same analysis of similarity between distributions as when investigating spatial effects, and discovered a block-like structure in the all-to-all JS divergence measurements across partner distance in certain contexts (Fig 5b). There appear to be two modes in courting male behavior depending on proximity to the female, and the drop off between these modes occurs at a distance of approximately *d_p_* = 4 mm. Surprisingly, the male-male context has a similar behavioral switch at this distance, although the effects are not as strong as in the courting context. The changes to the female behavioral density as a function of partner distance are more subtle in either female context, and suggest that social behavior in females is not as dependent on these simple variables.

In order to build models of social behavior, we must consider whether the individual preferences of animals within a pairing affect the behavior of both individuals. We first calculated the correlation coefficient between all pairs of behaviors performed by an individual animal in the single and paired contexts (S1 Fig). The correlation analysis within individuals is related to how animals distribute their time between different behaviors. There are several consistent trends across all individuals, such as positive correlations between crawling and locomotion, which simply means that individuals that tend to walk often also tend to run more than their counterparts. Another similar finding is that individuals that perform wing-related behaviors often also perform more posterior grooming, likely because these behaviors are related and are typically performed one after the other through the hierarchical transition structure of fly behavior [44–47]. Other features of both lone and paired individuals include negative correlations between vastly different behaviors. For example, there is a strong negative correlation in all cases between crawling and idle behaviors, which accounts for the fact that flies that spend their more of their time moving will spend less time standing still.

**Fig 6:**
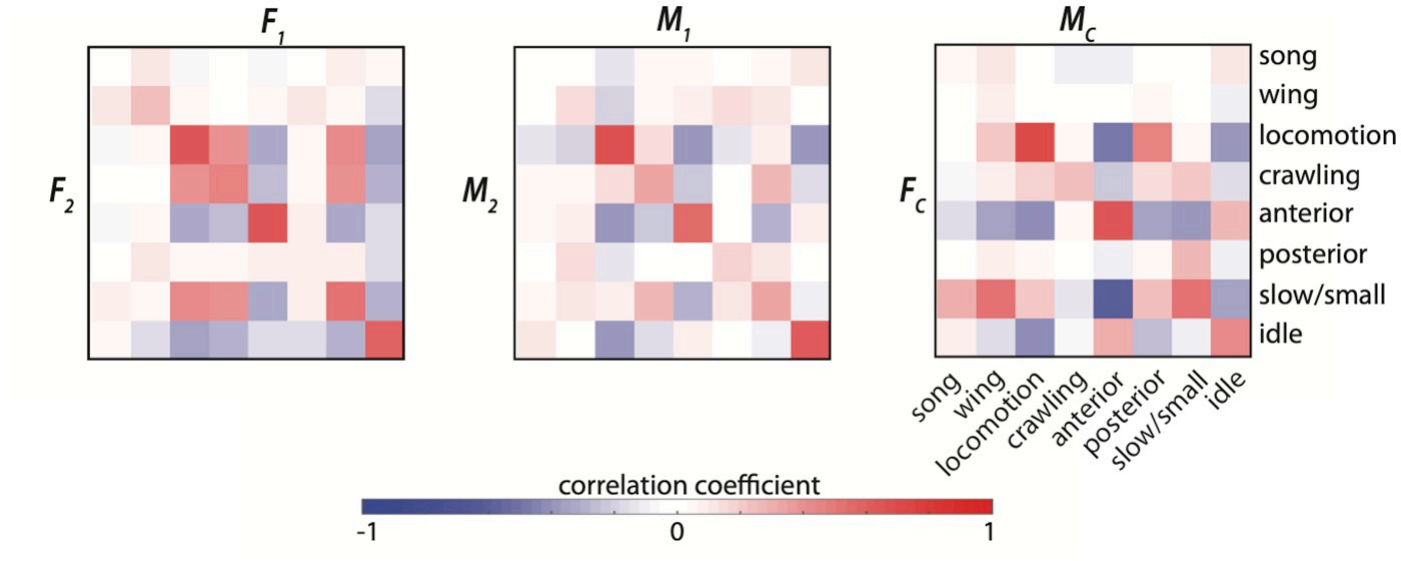
Correlation between behaviors performed by individuals within a pair. The correlation coefficient is displayed across each pairing for each possible pair of coarse-grained behaviors.

Correlations in behaviors between paired individuals illustrate how a social pairing influences the behavior of each present individual (Fig 6). We display the correlation values between coarse-grained behavioral densities of paired individuals and find that there are strong positive correlations across several behavioral pairings. We find that locomotion, anterior grooming, and idle behaviors are correlated in same-sex pairs of flies (correlation values between paired females are .64, .63, and .59, respectively, and correlation values between paired males are .68, .55, and .60, respectively). Courting flies also have a moderate to strong correlation for locomotion (*r* = .69) and anterior grooming (*r* = .65) within pairs, as well as other weaker off-diagonal correlations corresponding to courtship specific behaviors such as male wing behaviors and slow female movements (*r* = .52). These correlations represent up to a 20% increase in the frequency of an individual behavior (S2 Fig).

### Synchronization of instantaneous behaviors

**Fig 7:**
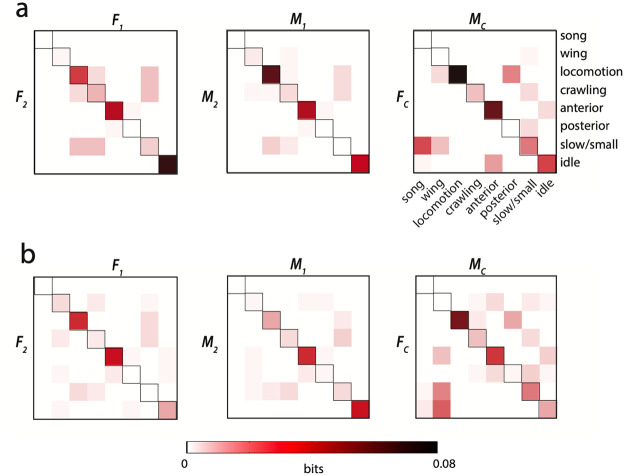
Partial mutual information between behaviors in paired animals. a) The partial mutual information for all data from each behavioral pairing in a paired context is calculated and positive values are displayed. b) The partial mutual information is calculated as in part (a) independently for each pairing in a given context, and the mean value is shown for each set of paired coarse behaviors. Only positive values are displayed. An enrichment on the diagonal of these matrices demonstrates synchronization of similar behaviors in time.

The correlation analysis above shows that flies will align their behavior when introduced to an arena together, however this does not tell us about the temporal structure of paired behavior. In order to address whether individuals change their behavior not just due to the proximity of the interaction partner but also the behavior it is performing, we looked for structure in the simultaneous behavior of paired flies. We calculated the mutual information (MI) between the ethograms of paired individuals and display the positive components that contribute to the total information in Fig 7a. We compared these measurements to the MI between non-interacting individuals by modeling the fly behavior as Markovian using the measured behavioral probability densities and transition matrices (S3 Fig). We find that in all three types of pairs there is a clear enrichment of information along the diagonal, which indicates that paired individuals perform the same behavior more often than is expected by underlying probability alone. This effect comes mainly from simultaneous locomotion, anterior grooming, and idle states, and varies according to pairing. Female-female pairs are particularly likely to remain idle together whereas male-male and courting pairs are more likely to locomote together, clear examples of Synchronized Behavior (Fig 1B).

The mutual information in the same-sex pairings is symmetric because each interaction is counted twice, once for each individual. The courting matrix considers the male and female behaviors separately. The non-negative partial mutual information in the first few columns means that there is some information about what the female is likely to be doing while the male is singing and performing wing motions, and this is most often idle behaviors, slow movements, and anterior grooming. When we sum over the partial mutual information values in each category, we can calculate the total mutual information for a particular context, summarized in Table 4.

**Table 4:** The sums across partial mutual information are calculated for each context from the matrices displayed in Fig 7 and 7b and from simulated data generated using a one-step MM with probability densities and transition data derived from behavioral sequences for each condition. All values are in bits.

Mutual Information Across Contexts			
Context	Total MI	Movie-Specific MI	Simulation MI
Female Pairs	9.54E-2	5.08E-2	5.54E-4
Male Pairs	8.95E-2	4.88E-2	7.67E-4
Courting Pairs	2.12E-1	1.10E-1	9.15E-4

When we calculate the mutual information (MI) on a movie-by-movie basis (Fig 7B), and consider only the underlying probability for each individual instead of all individuals in that context, we find that the information content is somewhat diminished across each of the contexts. This indicates that paired individuals have some mutual behavioral effect that is not simply related to their simultaneous actions. The enrichment in the simultaneous locomotion and simultaneous anterior entries informs us that even within a pairing, these behaviors are more likely to be performed together than by chance. The reduction in paired male simultaneous locomotion and paired female simultaneous idle states, however, indicates that this effect in the combined MI calculation is due to a synchrony effect on an experimental, and not temporal, scale. In other words, two females placed together may both become more idle for the course of a recording, but not necessarily synchronize the time spent idle in the way that occurs with other simultaneous behaviors. The same is true for males particularly prone to locomotion.

Histograms of partial mutual information (PMI) for several synchronized behaviors show that not all pairings contribute to positive non-zero MI values (S4 Fig). Courtship pairings with high PMI values for synchronous locomotion indicate times where males spent a long period of time chasing females, but even this interaction was seen in only some recordings. Similarly, each PMI distribution shows that many pairings, even when pooled data shows an effect, did not individually show synchronization.

**Fig 8:**
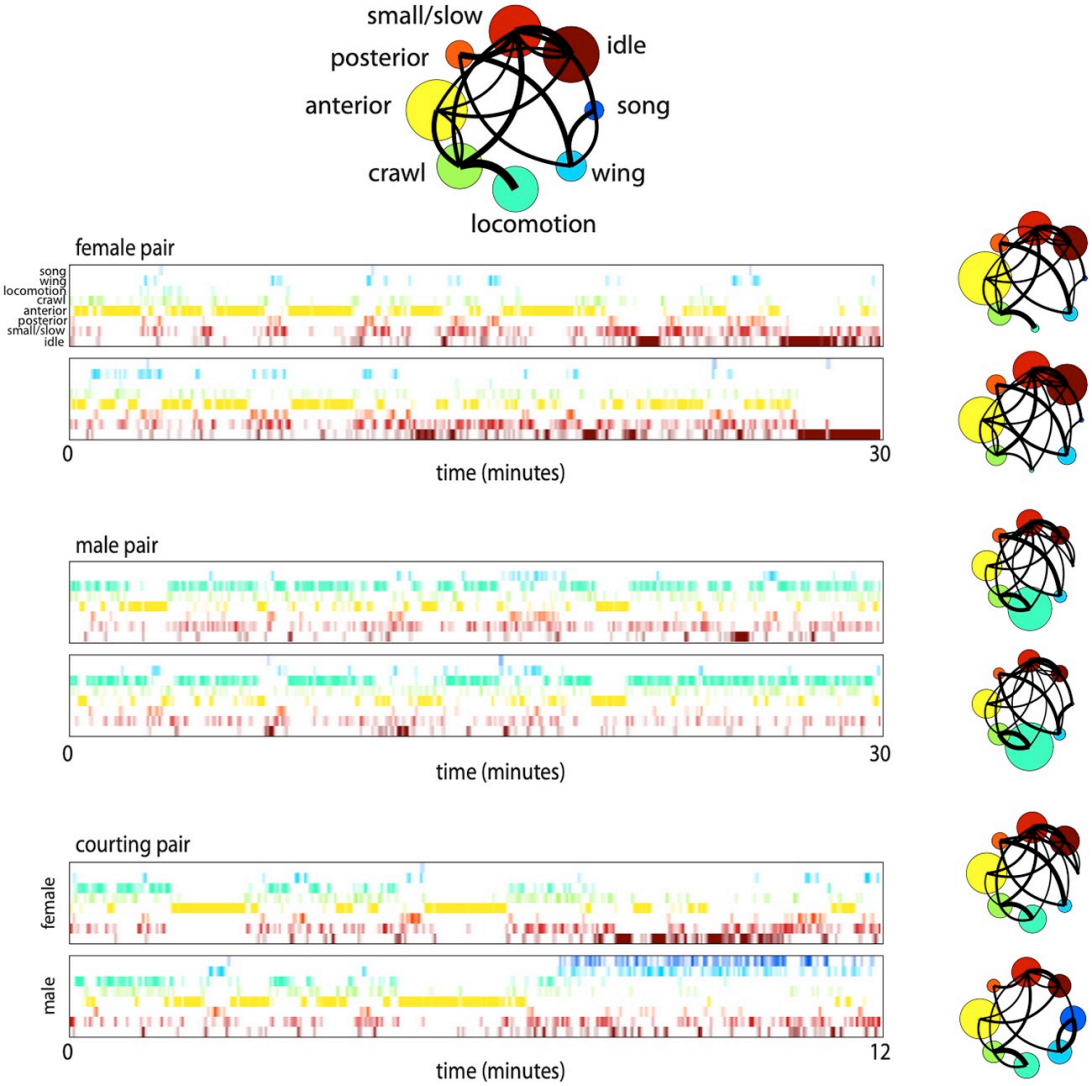
Sample ethograms and behavioral densities for fly pairs during simultaneous behavior. Ethograms for pairs of flies in each of the paired conditions are shown along with coarse behavioral densities for each individual. Colors and ordering used correspond to the coarse behavioral labels introduced in Fig 3 and replicated at the top for reference. Samples were chosen from experiments in the top quartile of synchronization. The switch to song behaviors in the male in the courtship example is representative of courtship samples where copulation was reached before the end of recording.

We illustrate the synchronization of behavior using ethograms of paired individuals that were in the top quartile of synchronization by correlation and mutual information metrics in Fig 8. The same-sex pairings display synchronization of anterior grooming, posterior grooming (which sometimes appears as a combination of wing movements and posterior grooming) and locomotion. Additionally, the overall densities of behavior look very similar within pairs, corresponding to the correlated behaviors described in Fig 6. For comparison, several ethograms from lone individuals are displayed in the same way (S6 Fig). The courtship example here is demonstrative of many successful courtship pairings, where grooming and locomotion may be synchronized early on, followed by the introduction of male song and an increase of slow female behaviors.

One possible explanation for the synchronization we observe based on MI values is that one individual will be startled or begin running based on the activity of the other individual sharing the arena. This could be based on visual cues that do not necessitate the presence of another fly, but instead simply any moving object. After exclusion of time points where either individual in an experiment was moving quickly (threshold = .4*mm*/*s*) we find that the positive distribution of PMIs indicating simultaneous anterior movements is not diminished (S5 Fig). This means that even within time spent standing still and performing only small movements, individuals spent more time than expected jointly performing grooming of their heads and antennae than expected by assuming independence.

**Fig 9:**
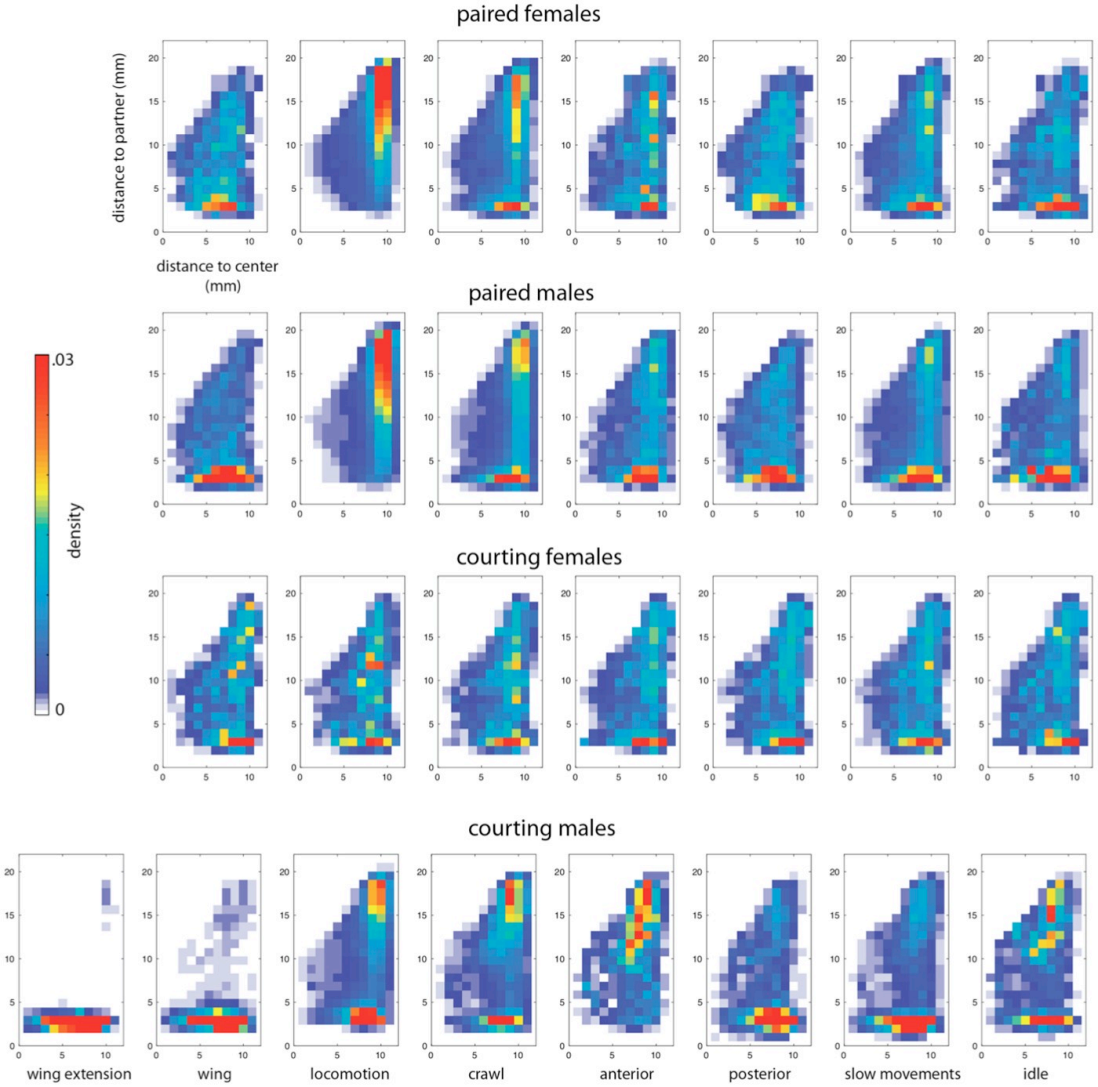
Location in the arena a behavior is performed depends on context. For each of eight behaviors (columns) for each paired context (rows), the 2-dimensional histogram of residence time in each paired context is broken down by distance from the center of the arena (x-axis) and distance to the interaction partner (y-axis). Each heat map summarizes where an individual was in the arena and relation to the interaction partner when performing a particular behavior.

While there are clear trends for preference of positioning between animals in a small arena (Fig 2), we also find that the preferential execution of different behaviors depends on an individual’s location in the arena and especially distance to its partner. We visualize these preferences given each of eight coarse behaviors in Fig 9. We observe differences between the behavior of flies between contexts that indicate different aspects of paired behaviors. Females in a courtship condition show few distinctions between behavioral preference, whereas a courting male shows a clear preference for different behaviors based on distance to his courtship target. Courting males differ from males in a same-sex pairing in several interesting ways in particular: they run while near their courtship target (chasing), and perform anterior movements as well as idle behavior more rarely when near the interaction partner. These preferences, coupled with a propensity to perform wing extension and wing motions (song) when near the female result in canonical courtship behavior.

## Discussion

Here, we investigate the structure of fruit fly social behavior at several scales, paying particular attention to the differences between paired and lone flies. We find that being in a social setting has a predictable effect on what an animal does that depends on the specific context. Unpaired animals respond strongly to the arena geometry and these effects persist in paired contexts. On top of this geometric modulation, the distribution of behaviors performed by an individual changes with the distance between animals and the difference in their headings. Our methods utilize new computer vision methods that can address the entire behavioral repertoire [48–50] and find changes across the spectrum of behaviors including locomotion and grooming.

We find evidence for three different ways in which social interactions modulate fly behavior (Fig 1). By examining the MI between the behaviors of paired animals, we find multiple examples in which the general behavior of an individual is altered by the presence of a partner (Fig 1C). Surprisingly, we also find evidence for the synchronization of behaviors between the individuals (Fig 1B), evidenced both by measurement of the MI and through examination of paired ethograms. Finally, we observed the introduction of song in males when in the presence of a female, a behavior that the male never performs when alone or when paired with another male (Fig 1D).

The use of behavioral clustering techniques that automatically produce labels for large amounts of data have allowed identification of other more subtle types of interactions [51–53]. We see a shift in behavioral density not only when pooling data over many individuals (Fig 3) but also within individuals experiencing different social situations (Fig 6). The correlation coefficients of certain behaviors (namely anterior-anterior, locomotion-locomotion, and idle-idle) between pairings of individuals within a given pairing indicate that each paired set of flies is unique but on average pairs tend to adjust or match their behaviors throughout an interaction. Mutual information analysis on pooled and individual interaction experiments confirms this matching, and indicates an even more precise temporal component of behavioral synchronization. Even by excluding data points where either individual was moving within the experimental chamber, we find that individuals perform anterior movements together more often than expected when assuming independence.

These aspects of behavioral organization offer an explanation for how more complex, and potentially collective, social behaviors are constructed [38, 39, 54]. Groups of flies may use synchronization in social situations to successfully navigate new environments by leveraging information from their con-specifics [55]. Some social imperatives, such as that of the male to chase and sing to the female, may outweigh these subtle effects. One aspect of the synchronization we observe is that it is inconsistent across pairs even within the same context. The stochastic nature of behavior may balance organizational principles and lend to exploration and variation. Social interaction lends itself to heightened states of motivation or arousal, and the quantitative approach presented here is one way to discover and characterize the behavioral tuning that accompanies these latent states [56, 57]. Given a way to identify these potentially subtle yet distinct behavioral effects, we may be able to begin linking these distributed effects to their genetic and neural bases [58, 59].

## Supporting information

S1 Fig Correlations between coarse behaviors across contexts. The behavioral density given eight coarse behaviors is calculated for each individual in a given context, and the correlation coefficient between all pairs of behaviors over all individuals in specified context is displayed. A higher correlation coefficient corresponds to a set of behaviors that co-occur more frequently within individual experiments than expected by chance.
(TIF)

S2 Fig Behavioral coupling across paired individuals. a) The fold change refers to the deviation in fraction of time paired individuals spent performing a set of behaviors simultaneously from the expected probability under the assumption of independence. b) The enrichment in the amount of time spend performing a set of behaviors simultaneously illustrates how much time individuals spent performing a set of simultaneous behaviors above expectation given the combined length of movies in an experiment.
(TIF)

S3 Fig Simulated and real joint distributions of behavior. a) The simulated non-interacting joint distribution is found by assuming independence between behaviors performed simultaneously in a given pairing. b) The real joint distribution of behaviors performed simultaneously between individuals in a given context.
(TIF)

S4 Fig Partial mutual information distributions across experiments. The distribution of partial mutual information values for (a) simultaneous locomotion, (b) simultaneous anterior movements, and (c) simultaneous idle behavior is shown for each of the three paired contexts.
(TIF)

S5 Fig Partial mutual information given small velocity. The partial mutual information distributions are calculated across each context for (a) simultaneous anterior movements and (b) simultaneous idle behavior after exclusion of all time points where either of the paired individuals is moving at a velocity above .4*mm*/*s*.
(TIF)

S6 Fig Sample ethograms and behavioral densities for lone flies. Ethograms for several isolated flies of each sex as well along with the associated coarse behavioral densities demonstrate the variety of behavior across individuals.
(TIF)

S1 Video Behavior_left_wing_song.mov. Examples of left wing song produced by many individuals, randomly sampled after labeling from behavioral map.
(MOV)

S2 Video Behavior_right_wing_song.mov. Examples of left wing song produced by many individuals, randomly sampled after labeling from behavioral map.
(MOV)

S3 Video Behavior_anterior_groom.mov. Examples of anterior groom produced by many individuals, randomly sampled after labeling from behavioral map.
(MOV)

S4 Video Behavior_fast_locomotion.mov. Examples of fast locomotion produced by many individuals, randomly sampled after labeling from behavioral map.
(MOV)

S5 Video Example_paired_female_anterior_groom.mov. Example of synchronized anterior grooming produced by paired females.
(MOV)

S6 Video Example_paired_female_anterior_groom_2.mov. Example of synchronized anterior grooming produced by paired females.
(MOV)

S7 Video Example_paired_male_anterior_groom.mov. Example of synchronized behaviors produced by paired individuals.
(MOV)

S8 Video Example_paired_male_anterior_groom_2.mov. Example of synchronized anterior grooming produced by paired males.
(MOV)

S9 Video Example_courtship_pairing_posterior_groom.mov. Example of synchronized posterior grooming produced by individuals in a courtship pairing.
(MOV)

S10 Video Example_courtship_pairing_anterior_groom.mov. Example of synchronized anterior grooming produced by individuals in a courtship pairing.
(MOV)

## References

[1] Tinbergen N. On aims and methods of ethology. Ethology. 1963;20(4):410–433.

[2] Morris D. Patterns of reproductive behaviour. 1970;.

[3] Lorenz K. On aggression. Psychology Press; 2002.

[4] Giuggioli L, Potts JR, Rubenstein DI, Levin SA. Stigmergy, collective actions, and animal social spacing. Proceedings of the National Academy of Sciences. 2013;110(42):16904–16909. 10.1073/pnas.1307071110 PMC3801015 24082100

[5] Bialek W, Cavagna A, Giardina I, Mora T, Pohl O, Silvestri E, et al Social interactions dominate speed control in poising natural flocks near criticality. Proceedings of the National Academy of Sciences. 2014;111(20):7212–7217. 10.1073/pnas.1324045111 PMC4034227 24785504

[6] Durisko Z, Kemp R, Mubasher R, Dukas R. Dynamics of social behavior in fruit fly larvae. PLoS One. 2014;9(4):e95495 10.1371/journal.pone.0095495 24740198 PMC3989340

[7] Ni R, Ouellette N. Velocity correlations in laboratory insect swarms. The European Physical Journal Special Topics. 2015;224(17-18):3271–3277. 10.1140/epjst/e2015-50077-5

[8] Louis M, de Polavieja G. Collective Behavior: Social Digging in *Drosophila* Larvae. Current Biology. 2017;27(18):R1010–R1012. 10.1016/j.cub.2017.08.023 28950082

[9] Dombrovski M, Poussard L, Moalem K, Kmecova L, Hogan N, Schott E, et al Cooperative Behavior Emerges among *Drosophila* Larvae. Current Biology. 2017;27(18):2821–2826. 10.1016/j.cub.2017.07.054 28918946

[10] Ramdya P, Schneider J, Levine JD. The neurogenetics of group behavior in *Drosophila melanogaster*. Journal of Experimental Biology. 2017;220(1):35–41. 10.1242/jeb.141457 28057826

[11] Benzer S. From the gene to behavior. Jama. 1971;218(7):1015–1022. 10.1001/jama.1971.03190200047010 4942064

[12] Sokolowski MB. *Drosophila*: genetics meets behaviour. Nature Reviews Genetics. 2001;2(11):879 10.1038/35098592 11715043

[13] Censi A, Straw AD, Sayaman RW, Murray RM, Dickinson MH. Discriminating external and internal causes for heading changes in freely flying *Drosophila*. PLoS computational biology. 2013;9(2):e1002891 10.1371/journal.pcbi.1002891 23468601 PMC3585425

[14] Simon AF, Chou MT, Salazar ED, Nicholson T, Saini N, Metchev S, et al A simple assay to study social behavior in *Drosophila*: measurement of social space within a group 1. Genes, Brain and Behavior. 2012;11(2):243–252. 10.1111/j.1601-183X.2011.00740.x PMC3268943 22010812

[15] Schneider J, Levine J. Automated identification of social interaction criteria in *Drosophila melanogaster*. Biology letters. 2014;10(10):20140749 10.1098/rsbl.2014.0749 25354920 PMC4272216

[16] Eyjolfsdottir E, Branson S, Burgos-Artizzu XP, Hoopfer ED, Schor J, Anderson DJ, et al. Detecting social actions of fruit flies. In: European Conference on Computer Vision. Springer; 2014. p. 772–787.

[17] Gautrais J, Ginelli F, Fournier R, Blanco S, Soria M, Chaté H, et al Deciphering interactions in moving animal groups. Plos computational biology. 2012;8(9):e1002678 10.1371/journal.pcbi.1002678 23028277 PMC3441504

[18] Katz Y, Tunstrøm K, Ioannou CC, Huepe C, Couzin ID. Inferring the structure and dynamics of interactions in schooling fish. Proceedings of the National Academy of Sciences. 2011;108(46):18720–18725. 10.1073/pnas.1107583108 PMC3219116 21795604

[19] Herbert-Read JE, Perna A, Mann RP, Schaerf TM, Sumpter DJ, Ward AJ. Inferring the rules of interaction of shoaling fish. Proceedings of the National Academy of Sciences. 2011;108(46):18726–18731. 10.1073/pnas.1109355108 PMC3219133 22065759

[20] Coen P, Murthy M. Singing on the fly: sensorimotor integration and acoustic communication in *Drosophila*. Current opinion in neurobiology. 2016;38:38–45. 10.1016/j.conb.2016.01.013 26874218 PMC6846365

[21] Coen P, Xie M, Clemens J, Murthy M. Sensorimotor transformations underlying variability in song intensity during *Drosophila* courtship. Neuron. 2016;89(3):629–644. 10.1016/j.neuron.2015.12.035 26844835 PMC5047376

[22] Stowers JR, Hofbauer M, Bastien R, Griessner J, Higgins P, Farooqui S, et al Virtual reality for freely moving animals. Nature methods. 2017;14(10):995 10.1038/nmeth.4399 28825703 PMC6485657

[23] Clemens J, Coen P, Roemschied F, Pereira T, Mazumder D, Pacheco D, et al Discovery of a new song mode in *Drosophila* reveals hidden structure in the sensory and neural drivers of behavior. bioRxiv. 2017; p. 221044. 10.1016/j.cub.2018.06.011 PMC6830513 30057309

[24] Klibaite U, Berman GJ, Cande J, Stern DL, Shaevitz JW. An unsupervised method for quantifying the behavior of paired animals. Physical biology. 2017;14(1):015006 10.1088/1478-3975/aa5c50 28140374 PMC5414632

[25] Zentall TR. Action imitation in birds. Animal Learning & Behavior. 2004;32(1):15–23. 10.3758/BF03196003 15161137

[26] Zentall TR. Imitation: definitions, evidence, and mechanisms. Animal cognition. 2006;9(4):335–353. 10.1007/s10071-006-0039-2 17024510

[27] Iacoboni M. Imitation, empathy, and mirror neurons. Annual review of psychology. 2009;60:653–670. 10.1146/annurev.psych.60.110707.163604 18793090

[28] Rizzolatti G, Craighero L. The mirror-neuron system. Annu Rev Neurosci. 2004;27:169–192. 15217330 10.1146/annurev.neuro.27.070203.144230

[29] Welty JC. Experiments in group behavior of fishes. Physiological Zoology. 1934;7(1):85–128. 10.1086/physzool.7.1.30151215

[30] Allee WC, et al. Animal aggregations. 1931;.

[31] Tolman CW. Social facilitation of feeding behaviour in the domestic chick. Animal Behaviour. 1964;12(2-3):245–251. 10.1016/0003-3472(64)90008-9 5882808

[32] Tinette S, Zhang L, Robichon A. Cooperation between *Drosophila* flies in searching behavior. Genes, Brain and Behavior. 2004;3(1):39–50. 10.1046/j.1601-183x.2003.0046.x 14960014

[33] Bates LA, Byrne RW. Imitation: what animal imitation tells us about animal cognition. Wiley Interdisciplinary Reviews: Cognitive Science. 2010;1(5):685–695. 26271653 10.1002/wcs.77

[34] Bloch G, Herzog ED, Levine JD, Schwartz WJ. Socially synchronized circadian oscillators. Proceedings of the Royal Society B: Biological Sciences. 2013;280(1765):20130035 10.1098/rspb.2013.0035 PMC3712435 23825203

[35] Connolly K. The social facilitation of preening behaviour in *Drosophila melanogaster*. Animal Behaviour. 1968;16(2-3):385–391. 10.1016/0003-3472(68)90023-7 5674242

[36] Byrne RW. Animal imitation. Current Biology. 2009;19(3):R111–R114. 10.1016/j.cub.2008.11.027 19211046

[37] Pasquaretta C, Battesti M, Klenschi E, Bousquet CA, Sueur C, Mery F. How social network structure affects decision-making in *Drosophila melanogaster*. Proceedings of the Royal Society B: Biological Sciences. 2016;283(1826):20152954 10.1098/rspb.2015.2954 PMC4810861 26936247

[38] Alisch T, Crall JD, Kao AB, Zucker D, de Bivort BL. MAPLE (modular automated platform for large-scale experiments), a robot for integrated organism-handling and phenotyping. Elife. 2018;7:e37166. 30117804 10.7554/eLife.37166 PMC6193762

[39] Versace E, Caffini M, Werkhoven Z, de Bivort BL. Individual, but not population asymmetries, are modulated by social environment and genotype in *Drosophila melanogaster*. Scientific reports. 2020;10(1):1–13. 32161330 10.1038/s41598-020-61410-7 PMC7066193

[40] Berman GJ, Choi DM, Bialek W, Shaevitz JW. Mapping the stereotyped behaviour of freely moving fruit flies. Journal of The Royal Society Interface. 2014;11(99):20140672 10.1098/rsif.2014.0672 PMC4233753 25142523

[41] Lin J. Divergence measures based on the Shannon entropy. IEEE Transactions on Information theory. 1991;37(1):145–151. 10.1109/18.61115

[42] Valente D, Golani I, Mitra PP. Analysis of the trajectory of *Drosophila melanogaster* in a circular open field arena. PloS one. 2007;2(10):e1083 10.1371/journal.pone.0001083 17957265 PMC2031922

[43] Shorey H, Bartell R. Role of a volatile female sex pheromone in stimulating male courtship behaviour in *Drosophila melanogaster*. Animal behaviour. 1970;18:159–164. 10.1016/0003-3472(70)90085-0 5494760

[44] Berman GJ, Bialek W, Shaevitz JW. Predictability and hierarchy in *Drosophila* behavior. Proceedings of the National Academy of Sciences. 2016;113(42):11943–11948. 10.1073/pnas.1607601113 PMC5081631 27702892

[45] Szebenyi AL. Cleaning behaviour in *Drosophila melanogaster*. Animal Behaviour. 1969;17(4):641–651. 10.1016/S0003-3472(69)80006-0

[46] Seeds AM, Ravbar P, Chung P, Hampel S, Midgley FM Jr, Mensh BD, et al A suppression hierarchy among competing motor programs drives sequential grooming in *Drosophila*. Elife. 2014;3:e02951 10.7554/eLife.02951 25139955 PMC4136539

[47] Mueller JM, Ravbar P, Simpson JH, Carlson JM. *Drosophila melanogaster* grooming possesses syntax with distinct rules at different temporal scales. PLoS computational biology. 2019;15(6):e1007105 10.1371/journal.pcbi.1007105 31242178 PMC6594582

[48] Anderson DJ, Perona P. Toward a science of computational ethology. Neuron. 2014;84(1):18–31. 10.1016/j.neuron.2014.09.005 25277452

[49] Berman GJ. Measuring behavior across scales. arXiv preprint arXiv:171205784. 2017;.

[50] Brown AE, de Bivort B. The study of animal behaviour as a physical science. bioRxiv. 2017; p. 220855.

[51] Gomez-Marin A, Partoune N, Stephens GJ, Louis M. Automated tracking of animal posture and movement during exploration and sensory orientation behaviors. PloS one. 2012;7(8):e41642 10.1371/journal.pone.0041642 22912674 PMC3415430

[52] Egnor SR, Branson K. Computational analysis of behavior. Annual review of neuroscience. 2016;39:217–236. 10.1146/annurev-neuro-070815-013845 27090952

[53] Robie AA, Seagraves KM, Egnor SR, Branson K. Machine vision methods for analyzing social interactions. Journal of Experimental Biology. 2017;220(1):25–34. 10.1242/jeb.142281 28057825

[54] Schneider J, Atallah J, Levine JD. 3 One, Two, and Many—A Perspective on What Groups of *Drosophila melanogaster* Can Tell Us About Social Dynamics. Advances in genetics. 2012;77:59 10.1016/B978-0-12-387687-4.00003-9 22902126

[55] Ramdya P, Lichocki P, Cruchet S, Frisch L, Tse W, Floreano D, et al Mechanosensory interactions drive collective behaviour in *Drosophila*. Nature. 2015;519(7542):233–236. 10.1038/nature14024 25533959 PMC4359906

[56] Krupp JJ, Kent C, Billeter JC, Azanchi R, So AKC, Schonfeld JA, et al Social experience modifies pheromone expression and mating behavior in male *Drosophila melanogaster*. Current Biology. 2008;18(18):1373–1383. 10.1016/j.cub.2008.07.089 18789691

[57] Lo SC, Scearce-Levie K, Sheng M. Characterization of social behaviors in caspase-3 deficient mice. Scientific reports. 2016;6:18335 10.1038/srep18335 26783106 PMC4726076

[58] Kennedy A, Asahina K, Hoopfer E, Inagaki H, Jung Y, Lee H, et al. Internal states and behavioral decision-making: toward an integration of emotion and cognition. In: Cold Spring Harbor symposia on quantitative biology. vol. 79. Cold Spring Harbor Laboratory Press; 2014. p. 199–210. 10.1101/sqb.2014.79.024984 25948637

[59] Anderson DJ. Circuit modules linking internal states and social behaviour in flies and mice. Nature Reviews Neuroscience. 2016;17(11):692 10.1038/nrn.2016.125 27752072

