# Supplementary figures and images for: Paired fruit flies synchronize behavior: Uncovering social interactions in Drosophila melanogaster
Source: PLoS Comput Biol. 2020 Oct 6;16(10):e1008230. doi: 10.1371/journal.pcbi.1008230 (PMC7567355; doi:10.1371/journal.pcbi.1008230)

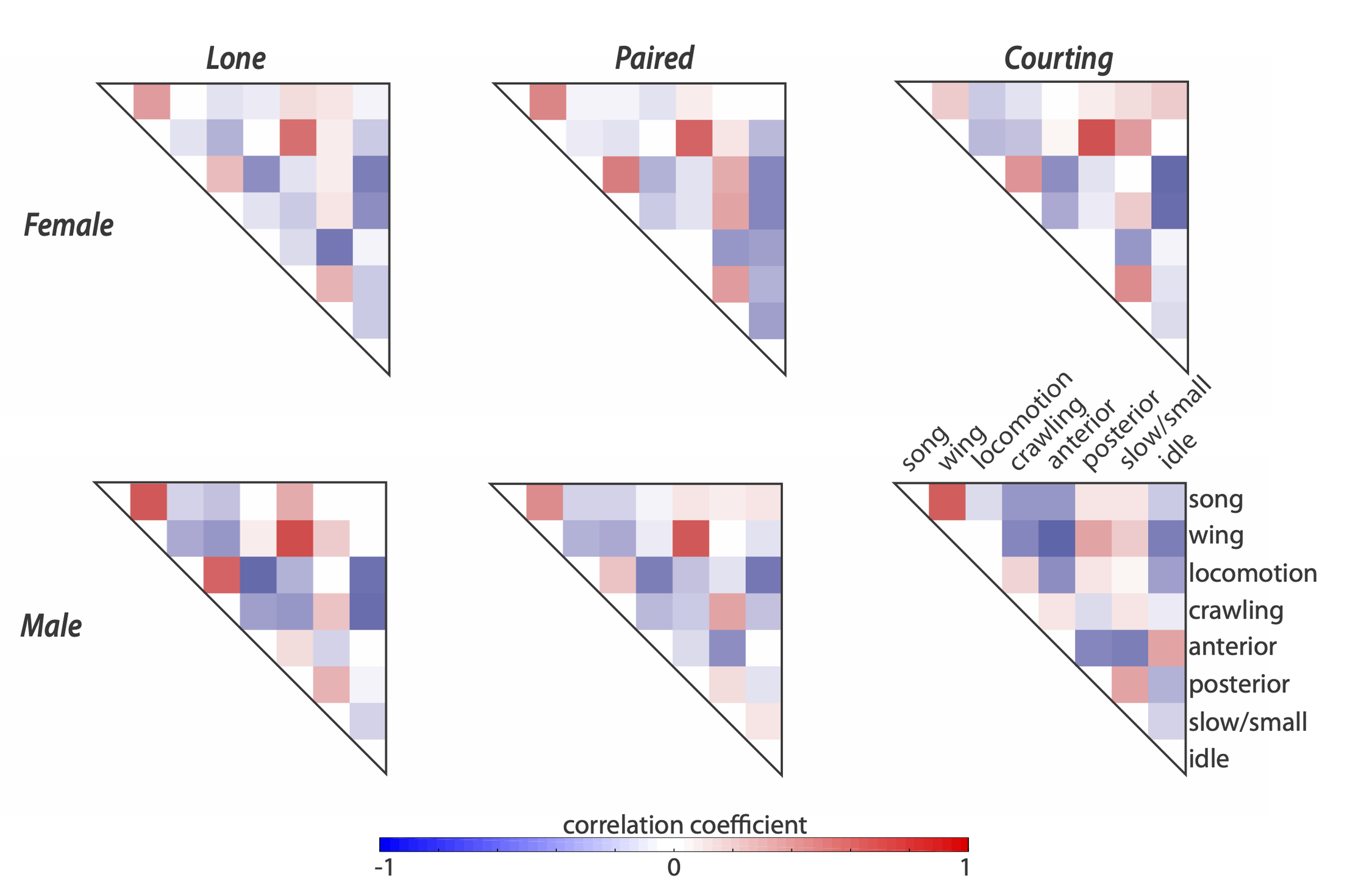

Supplement: S1 Fig — The behavioral density given eight coarse behaviors is calculated for each individual in a given context, and the correlation coefficient between all pairs of behaviors over all individuals in specified context is displayed. A higher correlation coefficient corresponds to a set of behaviors that co-occur more frequently within individual experiments than expected by chance. (TIF) [file pcbi.1008230.s001.tif]

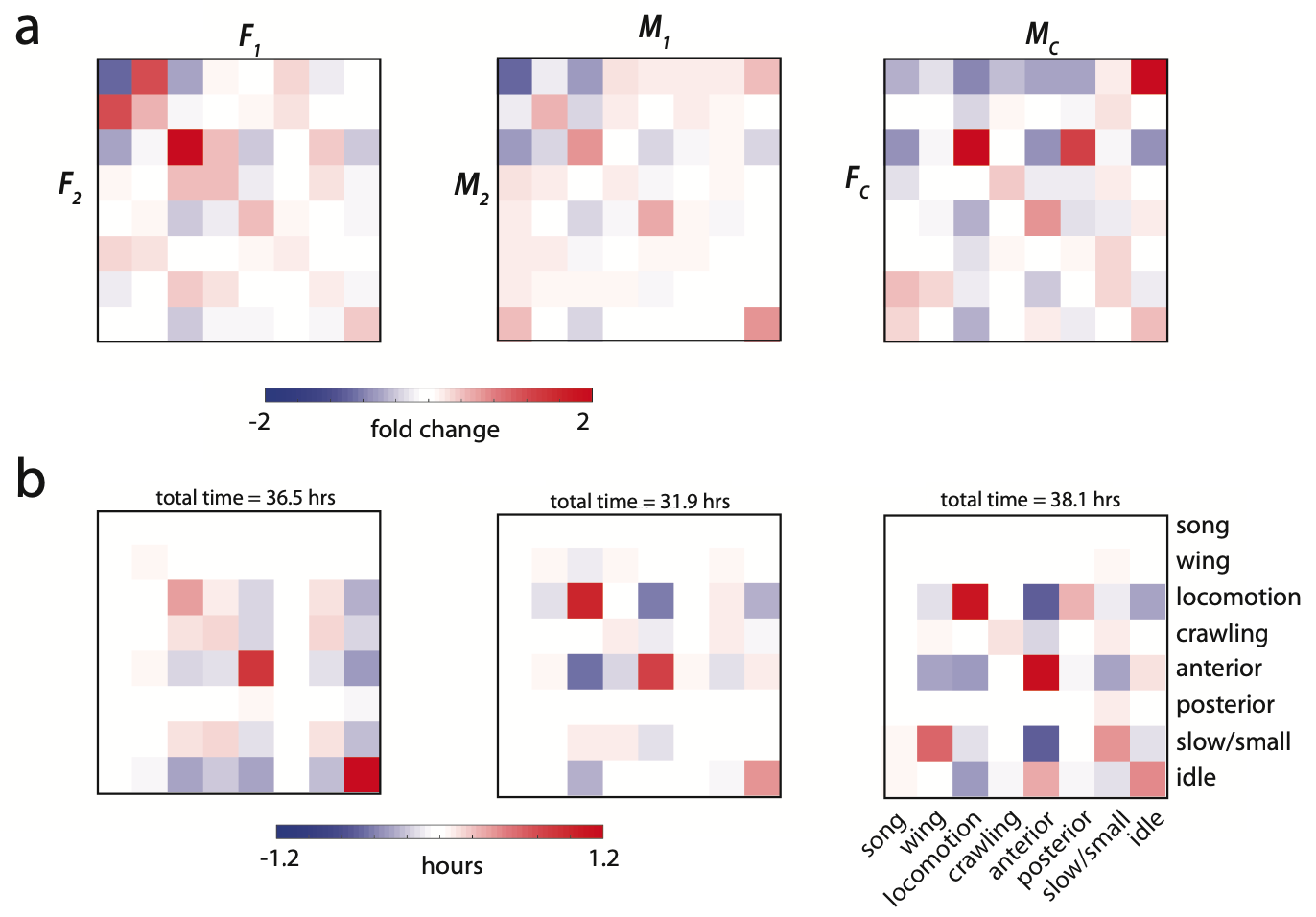

Supplement: S2 Fig — a) The fold change refers to the deviation in fraction of time paired individuals spent performing a set of behaviors simultaneously from the expected probability under the assumption of independence. b) The enrichment in the amount of time spend performing a set of behaviors simultaneously illustrates how much time individuals spent performing a set of simultaneous behaviors above expectation given the combined length of movies in an experiment. (TIF) [file pcbi.1008230.s002.tif]

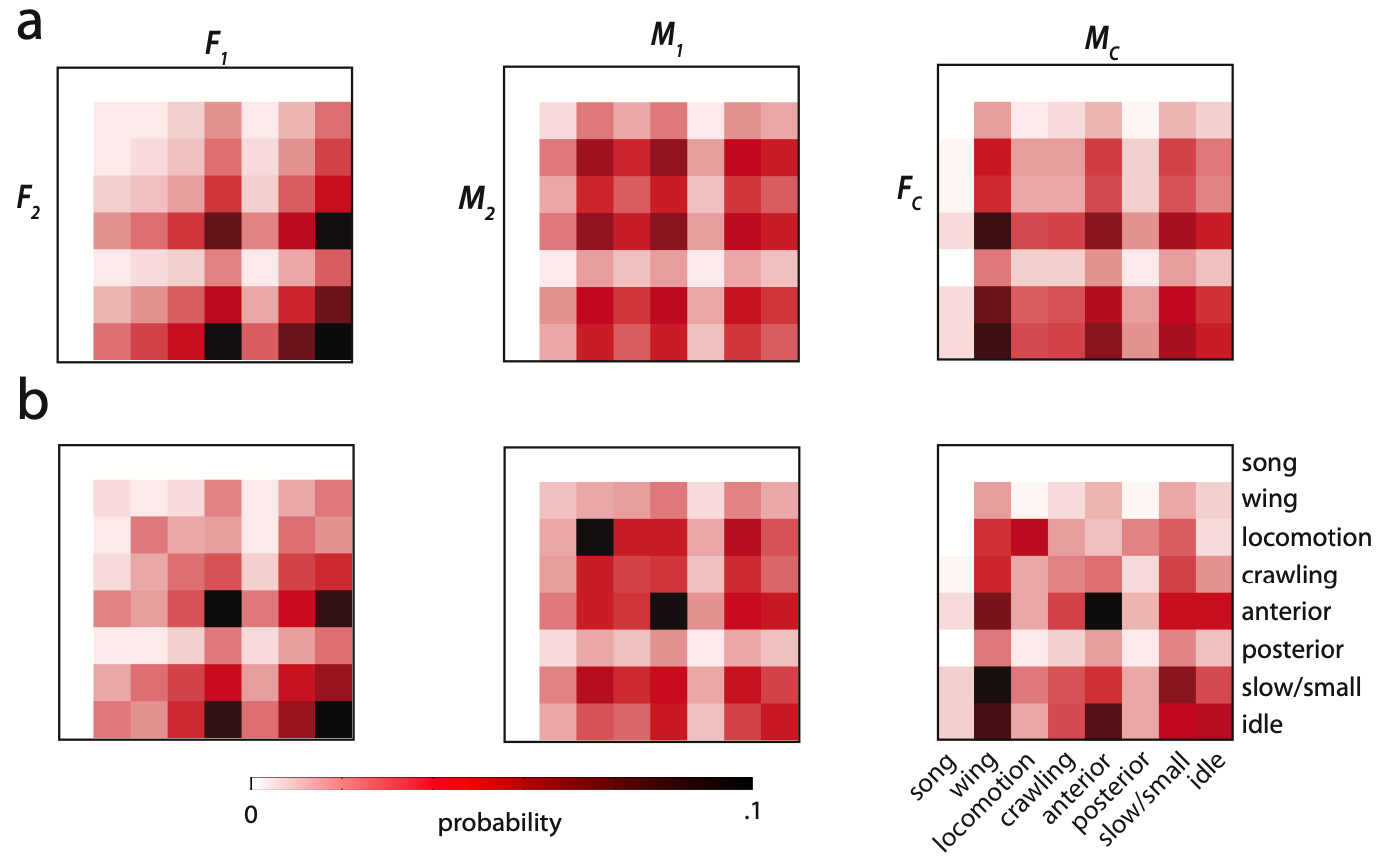

Supplement: S3 Fig — a) The simulated non-interacting joint distribution is found by assuming independence between behaviors performed simultaneously in a given pairing. b) The real joint distribution of behaviors performed simultaneously between individuals in a given context. (TIF) [file pcbi.1008230.s003.tif]

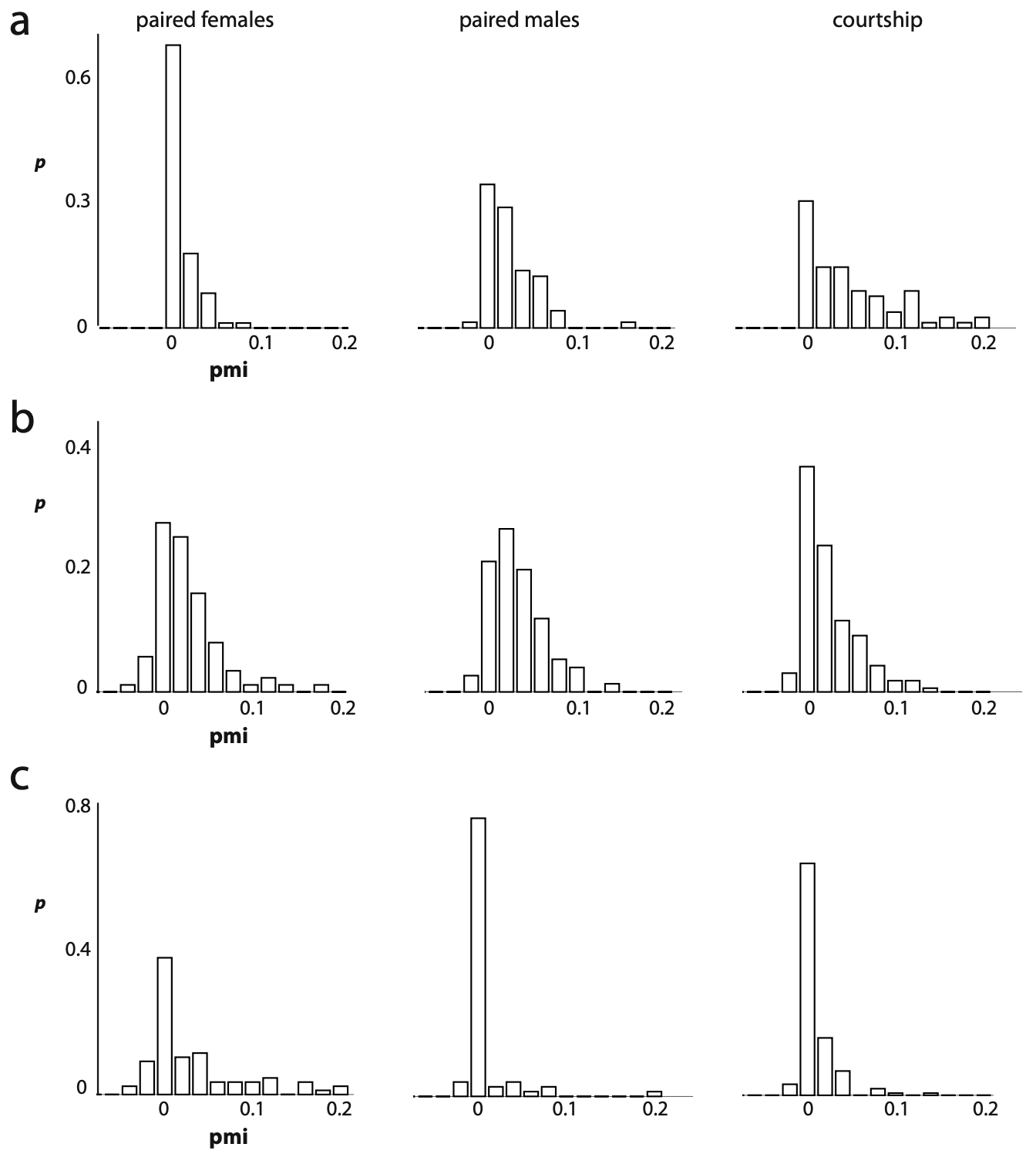

Supplement: S4 Fig — The distribution of partial mutual information values for (a) simultaneous locomotion, (b) simultaneous anterior movements, and (c) simultaneous idle behavior is shown for each of the three paired contexts. (TIF) [file pcbi.1008230.s004.tif]

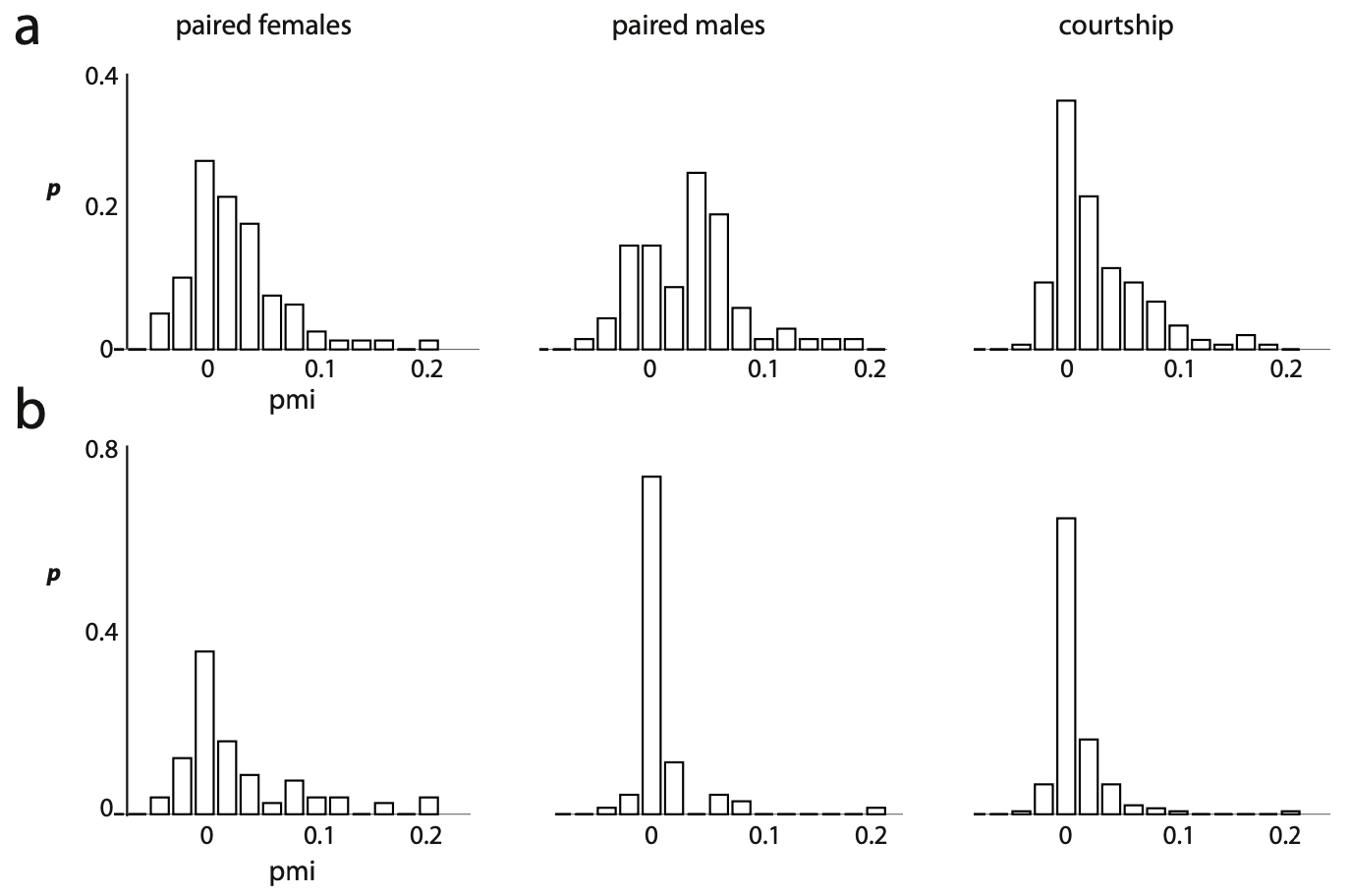

Supplement: S5 Fig — The partial mutual information distributions are calculated across each context for (a) simultaneous anterior movements and (b) simultaneous idle behavior after exclusion of all time points where either of the paired individuals is moving at a velocity above .4mm/s. (TIF) [file pcbi.1008230.s005.tif]

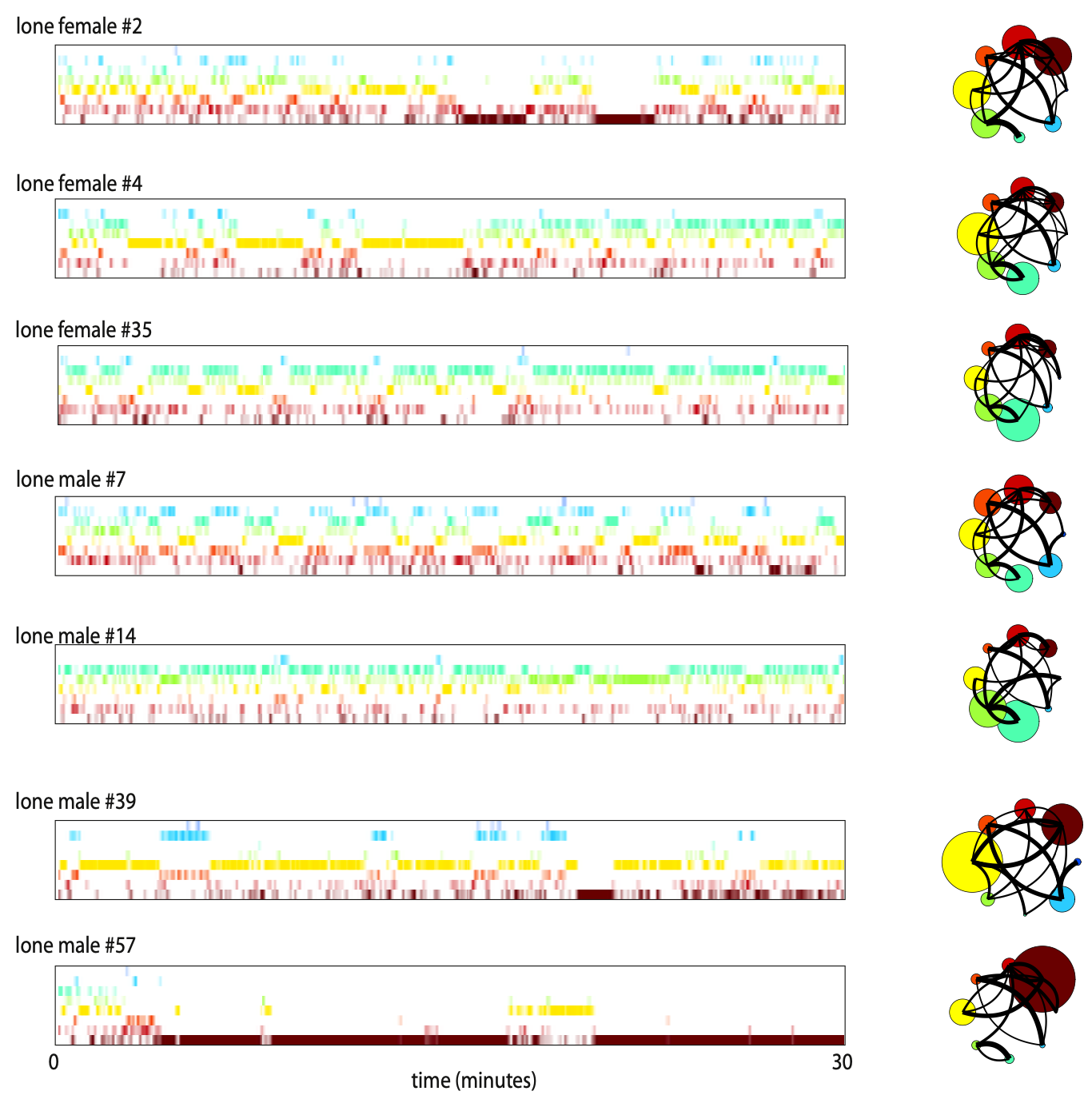

Supplement: S6 Fig — Ethograms for several isolated flies of each sex as well along with the associated coarse behavioral densities demonstrate the variety of behavior across individuals. (TIF) [file pcbi.1008230.s006.tif]
